# Analysing the digital transformation of the market for fake documents using a computational linguistic approach

**DOI:** 10.1016/j.fsisyn.2022.100287

**Published:** 2022-09-25

**Authors:** Clara Degeneve, Julien Longhi, Quentin Rossy

**Affiliations:** a Ecole des Sciences Criminelles, University of Lausanne, Switzerland; b CY Cergy Paris Université, France; c Institute of Digital Humanities, 33 Bd du Port, F-95000, Cergy-Pontoise, France; d Agora Lab, 33 Bd du Port, F-95000, Cergy-Pontoise, France; e Institut universitaire de France, 1 rue Descartes, F-75231, Paris, France

**Keywords:** Fake documents, Cryptomarket, Computational linguistic, Textometry, Language trace

## Abstract

The market for fake documents on the internet is a topic that has not yet been explored in depth, despite its importance in facilitating many crimes. This research explored the market of fake documents on the White House Market anonymous market with a computational linguistic methodology; more specifically using textometry. The textual corpus is composed of the data of the ads titles as well as the profiles of the sellers, which were analysed as traces of their online activities. We investigated how these remnants can help to answer general questions. What kinds of fake documents are sold? Can we distinguish types of sellers based on their selling activities or profiles? Can we link distinct vendors based on language trace similarities? The free software IRaMuTeQ was used to carry out the analysis. The results showed that the textometric methods have real potential in classification, highlighting the different products on the market, and grouping the sellers according to their offers.

## Highlights

• Analyse the market of fake documents on an online anonymous market.
• Explore the informative potential of computational linguistics to analyse language traces.
• Highlight the digital transformations of the market of fake documents to specific types of digital document.

## 1 Introduction

Identity documents are required for many everyday activities such as subscribing to a telephone service, taking out a loan from a bank, crossing borders, or buying alcohol. In addition to granting rights to their rightful holder, they can confer trust, authority, benefits, and responsibilities. This makes them highly attractive assets for individuals deprived of such benefits. Document fraud is thus a convenient solution, sometimes the only one, to get past identity checks and controls and access to the places or services sought [1]. But identity documents are not the only documents used for obtaining benefits and are thus not the only ones affected by forgeries. They are a particular type of ‘secure document’ such as travel documents, banknotes, or diplomas, which can be defined as a document giving legal or commercial function and value to the holder and having the property of allowing the confirmation of its veracity, validity, and authenticity as a genuine document [2]. This makes fake secure documents a hot product for the illicit market. This paper will refer to ‘fake documents’ as forgeries of identity document and secure documents.

The market for fake documents has found its way to extend to online marketplaces. The marketplace chosen for this research is the ‘White House Market’ (WHM) cryptomarket, still active at the moment of the study from January to June 2021. It was then one of the most active cryptomarkets on the Dark Web,^1^ until its closure in October 2021, with nearly five hundred thousand users and almost three thousand sellers.

We focus on the textual data present in HTML traces collected on the WHM cryptomarket. These traces can be apprehended from several forensic perspectives. The one we focus on is called by Renaut and colleagues (2017) the “language trace”. It is the remnant of an action [3,4] which is the writing of an illegal or litigious text by an author with an informative potential on its source, but also on the illicit activity itself. Language trace may result from illicit acts that can be committed through language, such as threats, defamation, or even an apology for terrorism [5]. In this study, we investigate language traces resulting from the publication of illegal ads posted by vendors to reconstruct their activities and get insight into the online market for fake documents. We investigate how these remnants can help to answer general questions: (1) “What kinds of fake documents are sold?”, (2) “Can we distinguish types of sellers based on their selling activities or profiles?”, (3) “Can we link distinct vendors based on language traces similarities?”.

The analysis of ‘words as traces’ in the forensic context raises many questions about the objectivity, reliability, and reproducibility of the methods used to analyse language traces. Since traces are more often than not considered as silent witnesses, considering words as traces is not obvious. From a methodological point of view, this research is thus based on computational linguistics, which, integrated with forensic science, is commonly called “forensic linguistics”. Seen as a particular field of applied linguistics, it is defined as “*a branch of linguistics which applies in the field of justice techniques from linguistics and phonetics for the analysis of evidence in court”* ([5]; p. 426 free translation). However, such a definition reduces the scope of the methods to the trial [6], whereas forensic science covers the exploitation of traces more broadly in policing [4]. Indeed, computational linguistics approaches can be exploited for global forensic purposes, such as authorship attribution [[7], [8], [9]], or the recognition and classification of illegal activities [10,11].

In this case study, textometric methods have been selected to recognise and classify illegal activities tasks. Textometry is based on “*the lexicon, that is the counting and distribution of words within the texts of a corpus, but also other levels of linguistic and textual description (morphosyntax, textual structures, etc.)*” [12]. The main interest in choosing this method is that it includes both a quantitative and a qualitative dimension. Indeed, textometry is based on a statistical analysis of textual data, but it integrates what [12] calls a “*back to the text*” step, where the scientist evaluates the results of the computational analysis by considering the surrounding context of the detected textual forms.

This paper is structured as follows: first, a review of the existing literature on the online market for fake documents is presented. Then, the research methodology and the different technical aspects are developed. Finally, the results are presented and discussed.

## 2 The online market for fake documents

The market for fake documents has extended to online marketplaces [[13], [14], [15]]. These online markets form a specific type of ‘virtual convergence settings’ where offenders (i.e. sellers and buyers) interact and leave traces [16,17]. They can take multiple forms, such as publicly accessible websites (e.g. online shops or platforms) or more private channels of communication, such as private groups on social media or instant messaging app. Because private settings are more difficult to study due to accessibility and ethical issues, this research focuses on a specific type of public setting: Online anonymous market present on the “.onion” darkweb relying on the TOR network, which is also known as a ‘cryptomarket’. A cryptomarket is an online marketplace on the darkweb, which is quite similar to regular e-commerce platforms. Sellers post their ads and payments are carried out by cryptocurrencies. These anonymous markets allow users to engage in illegal activities while limiting the risk of being checked by the authorities [18,19].

In addition to the reasons for accessibility, this choice to analyse a cryptomarket is based on three main reasons. First, online platforms bring together a variety of sellers and buyers, allowing for analysing the activity of multiple stockholders as a whole, whereas dedicated online shops selling fake documents appears to be quite rare [20]. Second, the tracking of dedicated online shops involves gathering heterogeneous data, whereas platforms have a unified internal structure. Finally, the choice of monitoring the “.onion” darkweb is adequate since illicit markets on the web are known to contain scams, while darkwebs give a higher level of anonymity.

Indeed, darkwebs such as “.onion”, which is recognised as the main one, concentrate illicit activities and in particular illicit markets. They offer a high degree of anonymity for both the manager of the websites and their users. They are not regulated by the DNS system of the ICANN, but are “Special-Use Domain Names” that are auto-regulated and self-authenticating since they are solely derived from cryptographic keys [21,22]. Moreover, the “.onion” darkweb is settled upon the TOR network which secures the content of communication through encryption and protect anonymity with the use of multiple intermediary nodes and a dedicated communication process known as the “onion routing” to exchange information between computers without directly exchanging identifying information such as IP addresses [23].

[24] have formalise the mechanism for the online selling of fake ID documents with a crime script. By analysing 19 sellers found both on the Clear and Dark Web, they identified four main steps:

- “P*recondition of potential customers”*: These are the arguments put forward by sellers to attract buyers, such as the possibility of travelling,
- “I*nitiation and entry into the market*”: Buyers can access markets via their browsers, sometimes after viewing advertisements that allow them to choose the seller. An initial contact then takes place between the buyer and the seller,
- “V*endor actualisation and doing of document creation*”: The buyer pays for the order after having outlined their requirements to the seller, who then proceeds to create the document. The seller then proceeds to create the document,
- “E*xit scripts of the customer and vendor*”: Once the transaction is done, contact is often broken between the seller and the buyer, except for those trying to build customer loyalty or offering order tracking.

This description of the process outlines two dimensions of investigation about the Market. The first one is related to the nature of the target of the transaction (i.e. the fake document). The questions are “what types of documents are buyers looking for” and “for what purposes”? The second dimension is related to the means of contact used to enter the market. The questions are “what are the means” and “how to detect and monitor online settings used”? Globally, there are still very few specific studies addressing these questions about the market for fake documents. This might probably be explained by the small proportion that fake documents represent among all other illicit products available on cryptomarkets.

According to the study of Baravalle and colleagues [13], that analyses the sale of fake documents on cryptomarkets, these products are much less prevalent than others, such as drugs, which account for 80% of the products for sale on the “*Agora*” cryptomarket (N = 30,680 products and sellers pages collected). By comparing ads for drugs and fake IDs on this platform, they determined that the market for fake IDs was more concentrated, with fewer sellers and ads than for drugs.

In his book, Akhgar and colleagues (2021) consider the fake identity document market within the “*fraud and counterfeit*” category of products that can be found on the Dark Web, among five other major product types. The description given is limited to “*Fraud and counterfeits – the document fraud, with the online trading of fraudulent, fake, stolen and counterfeited documents and cards, such as fake passports or identification cards and cloned and stolen credit cards or accounts, is emerging and one of the fastest-growing markets, in all types of criminal activities including terrorism. ‘Card shops’, for example, are one of the speciality markets in the Dark Web*.” [25]; p. 101).

In Mireault's MSc thesis (2016), fifty websites selling counterfeit documents on the web were analysed to describe their visibility, products sold and the sales process. The online stores appear to exploit online forms and emails as their preferred means of communication. They also favour payments by digital currency (e.g. Bitcoin), and international money transfers (Western Union and MoneyGram), which are well-known to be used by scammers. The main types of fake documents detected were a driver's license on 68% of the websites (n = 34), identity cards (28%, n = 14) and student cards (24%, n = 12). Passports, visas, residence and civil status documents were detected on 16% of websites (n = 8). Professional cards, diplomas, and fancy documents were sold on fewer sites (10%, n = 5).

On the darkweb, dedicated online shops selling fake documents appears to be quite rare. Laferrière and Décary-Hétu [20] identified 108 illicit online shops, but only 6 (5.5%) are dealing in fake documents. Much more websites appear to sell drugs (37%, n = 40) or carding credentials (31%, n = 34). No information on the products sold is detailed in this global study.

Bellido and colleagues (2017) investigated the acquisition mechanisms of fake documents to establish a state of the market. Using a keyword search on Bing, Yahoo, and Google browsers, as well as a more extensive search for new links contained in previously crawled pages, they obtained a total of 375 URLs, 357 distinct hostnames, and 223 identifiers. They determined the most common ways sellers make themselves visible to their buyers via different web spaces. Dedicated videos represent “*37% of the means of selling*”, publications on forums and blogs represent 27% of these methods, hidden TOR sites 19%, dedicated sites 12%, and evaluation and advice sites represent 5% of the means of selling. The authors also detailed the sales process by first determining the main motivations invoked by sellers to induce customers to buy a fake ID, as well as the main means of contact and ordering. Their results seem to show that, regardless of the distribution medium used, email is consistently found as a means of contact, even if it is not the most frequent. They then conducted a market analysis to see which products are the most sold and at what price. These parameters vary depending on the platforms used, but driver's licences seem to be the most commonly sold and cheapest document, compared to passports and ID cards. These results are consistent with the results found by [15].

## 3 Methodology

### 3.1 Dataset

The data used for this research was collected from the cryptomarket ‘White House Market’ (WHM). This cryptomarket, online from February 2019 to October 2021, was one of the major cryptomarkets in the Dark Web at the end of the study. Twenty crawls were performed from August 11, 2020 to March 11, 2021. The webpages of the advertisements and the sellers' profiles have been extracted for 83′516 distinct ads and 2′519 distinct vendor profiles (see Table 1). All parts of the collection process were based on open-source APIs and our own developments.

**Table 1:** Number of distinct vendors and ads for each section of the cryptomarket. The number of ads is counted based on distinct URLs of the ads, but also with the number of distinct product titles for each product since the product title might have changed over time.

Sections	Distinct Vendor Url	Distinct Product Url	Distinct Product Title
**Drugs**	2′296 (91.1%)	68′699 (82.3%)	82′618 (84%)
**Online Business***(excluding SSN/DOB/PII)*	183 (7.3%)	6′681 (8%)	7′294 (7.4%)
**Services***(excluding “Fake Documents”)*	163 (6.5%)	2′275 (2.7%)	2′343 (2.4%)
**Software**	85 (3.4%)	2′522 (3%)	2′606 (2.6%)
**Forgeries/Counterfeits**	81 (3.2%)	1′785 (2.1%)	1′841 (1.9%)
**Online Business > SSN/DOB/Other PII**	72 (2.9%)	384 (.5%)	445 (.5%)

**Services > Fake Documents (Digital)**	62 (2.5%)	772 (.9%)	801 (.8%)
**Services > Fake Documents (Physical)**	35 (1.4%)	331 (.4%)	343 (.3%)
**Defense/Counter Intel**	27 (1.1%)	76 (.1%)	84 (.1%)
**Total**	**2′519 (100%)**	**83′516 (100%)**	**98′375 (100%)**

The sections presented here have subsections. The subsections “*Fake Document (Digital)*” and “*Fake Document (Physical)”* are included in the section “*Services*”. As this study focuses on fake documents, those two subsections are treated separately from the rest, for a total of 1103 advertisements (1.3% of all ads) and 86 vendors (3.4% of all vendors).

### 3.2 Pretreatment

To carry out the textometric analysis, we chose to use the software IRaMuTeQ,^2^ a free software based on Python and R. It allows multiple statistical analysis and produces visualisations. It has been chosen for its ease of use and the available textometric methods.

To integrate the data into the software as corpus (i.e. a set of text units to be analysed), they have to fit with a particular format called “*Alceste*” [26]. First for the ads, each category is separated from the others and converted into a.txt document (UTF-8 encoding) containing the ad title, category and vendor's name. Every new text is introduced with four asterisks “****“. These are followed by the first information, here the name of the vendor, like “*_name1” and then the name of the corresponding category in the same format. These variables are called “*illustrative variables*”, which means that they are not part of the text analysed but are used to filter the dataset. The text submitted to textometric analysis is the title of the ad. The descriptions of the products in the ads have been tested in several analyses but didn't give sufficient results to be considered relevant and thus are excluded. The same process is used to prepare the corpus composed of the 86 vendors of fake documents, with their names and date of admission to White House Market as illustrative variables and their profiles for the textometric analysis.

In Table 2, it is possible to see that only 69 vendors are taken into account for the “*vendor_fakedoc*”. This can be explained by the fact that 17 vendors don't have any written profile. The corpora containing two categories are called “*mixed corpora*”. Section specific corpora are used to obtain monothematic sets to avoid replication of the initial structure of the sections [27].

**Table 2:** Description of all the corpora created from the data and integrated in IRaMuTeQ.

	Corpus	Description	Number of texts
Section specific	listing_defense	« Defense » section of the cryptomarket	88
	listing_drugs	« Drugs » section of the cryptomarket	85262
	listing_forgeries	« Forgeries » section of the cryptomarket	1894
	listing_onlinebusiness	« Online business » section of the cryptomarket	9056
	listing_services	« Services » section of the cryptomarket	2509
	listing_software	« Software » section of the cryptomarket	2883
	listing_fakedoc	« Fake Document Digital/Physical » subsections	1321
	listing_all_without_drugs	All listings except the drugs section	17751
	listing_all	All listings	103013
Mixed	listing_fakedoc/drugs	Combination of the “fakedoc” and “drugs” corpora	86583
	listing_fakedoc/forgeries	Combination of the “fakedoc” and “forgeries” corpora	3215
	listing_fakedoc/onlinebusiness	Combination of the “fakedoc” and “online business” corpora	10377
	listing_fakedoc/services	Combination of the “fakedoc” and “services” corpora	3830
	listing_fakedoc/software	Combination of the “fakedoc” and “software” corpora	4204
	listing_fakedoc/defense	Combination of the “fakedoc” and “defense” corpora	1409
	vendor_fakedoc	Fake documents vendors with a written profile on the cryptomarket	69

Since most of the texts analysed are written in English, the English dictionary is used. For the other parameters of the software, the default values are used.

All the texts are then lemmatised, i.e. all the forms are reduced, “*so that a conjugated verb can be reduced to its infinitive, plural and singular forms, masculine and feminine forms can be grouped together, and, more generally, forms corresponding to the same root with different inflections can be grouped together*” [28]; p. 867). The interest of this step is to group the main ‘forms’ and their derivatives under a single label to have a more robust statistical analysis.

The next paragraph describes the textometric methods used on the corpora.

### 3.3 Descending Hierarchical analysis (DHA)

Marpsat describes DHA as a method that allows one to “*give an account of the internal organisation of a discourse*” [26]; p. 1). After separating the forms thus obtained into two categories, the “*analysable forms*” (i.e. terms of the text taken into account during the analysis) and the “*illustrative forms*” [26]; p. 2) that having a purely descriptive value for the classes obtained from the analysable forms, the text is cut into segments. These are parts of the text of fixed size, often delimited by punctuation or special characters. These text segments are then grouped together to contain enough analysable forms for analysis. They constitute the context of the words. They are created automatically by the software (three lines) [27]. A “*lexical table*” [26]; p. 2) is then formed with the groups of segments in rows and the analysable forms in columns. Finally, the DHA is carried out, gathering the groups of segments into classes. The table values contain ‘1’ if the analysable shape is present in the segment group and ‘0’ if it is absent. The algorithm then produces a successive division of the groups into classes, first two, then two more from the largest and so on. The aim is to obtain clusters based on form frequencies representing “*lexical worlds*” [29] of the texts classified. They are traces of the own ‘world’ (i.e. discourse universe) of the reconstructed class [29]. They are reconstructed solely upon the forms (and segments) independently of any semantic interpretation.

DHA is performed automatically with IRaMuTeQ, and has been applied to the product's corpus “*listing_fakedoc*”. The program took into account 1187 texts over 1321. One hypothesis that could explain this exclusion of certain texts is that the software performs a pre-arbitration in the texts, if some are too heterogeneous compared to the rest of the corpus and are therefore excluded before the analysis. It has been applied to the mixed corpora too (see Table 2), to see which categories of products can be found with this method.

Once the DHA is done, several statistics are automatically performed. The number of occurrences of every studied form (i.e. a bag-of-word model) is used to examine each form in a concordance table. It allows observing the form in its original context (i.e. text segments) regarding the illustrative variables (i.e. the section to which the product belongs or the vendor for instance). Ads published in the wrong sections can thus be identified.

The analysis is finally performed with the “*listing_all_without_drugs*” corpus. The choice to remove all drug ads is made because there are too many drug ads compared to the rest of the products. Then the first five most frequent words of each class created with the DHA are compared with the classification made with the “*listing_fakedoc*” corpus.

### 3.4 Specificities and correspondence factor analysis (CFA)

CFA is a complementary analysis of DHA, which allows associating texts with variables. The DHA table is projected on the axis defined by chosen variables (e.g. the vendor id). It gives a graphical representation of the distance between the different groups according to the analysable forms [30]. CFA process a statistical analysis (in our case a hypergeometric law) based on the selected variable.

It is automatically generated successively to the DHA analysis applied to the “*listing_fakedoc*” corpus, showing the distance between the different classes found by the DHA, then to the mixed corpora. To find groups of vendors based on their catalogue and then based on their profile, CFA has been applied several times in succession to the corpora “*listing_fakedoc*” and “*vendor_fakedoc*”. It was produced using the name of the vendors as the variable. Before each new analysis, the vendors furthest from the core group (named “*outliers*”) were removed until no more outliers were detected. The groups of vendors were finally defined based on their position on the axes. Finally, the outliers were analysed separately to understand what makes them different from the main set of vendors.

### 3.5 Similarity analysis

This analysis aims to “*study the proximity and relationships between the elements of a set, in the form of trees*” [31]; p. 3). The links between forms are visualised with a graph model. Nodes are forms and links are based on their presence in the same text, which leads to a typical cooccurrence graph. Since the readability and interpretability of a cooccurrence graph are complex due to the multicity of links between nodes, the maximum spanning tree is used to visualise the results [27].

Similarity analysis is applied to the “*listing_fakedoc*” corpus, conserving the default settings of IRaMuTeQ. The visualisation of the result has been made using the “*yEd*” software,^3^ IRaMuTeQ providing only a “*.png*” image of the graph. The “natural clusters” algorithm detects clusters of words where each word is only in one group, maximising the number of edges within it and minimising the number of edges between other groups [32].

### 3.6 Ethical consideration

The collection process relies on online open data gathered with ad hoc web-crawling and web-scraping technologies. The cryptomarket of interest can be considered as a public space regarding the massive number of users and sellers, with data available for every user. Access to the website is conditioned by account creation, but anybody can create one without any condition. To respect privacy, all the vendor's names were anonymised, and no other identifying information was used during the study. All the analyses were based on the texts, and the results are presented so that no link can be established with the virtual identity of the sellers. The vendor's profiles were crawled but are not presented in the results. The collected data is intended exclusively for research purposes and cannot be used in any way that could be harmful to the users since no personal data is shared.

## 4 Results

### 4.1 Classification of fake documents

**Fig. 1:**
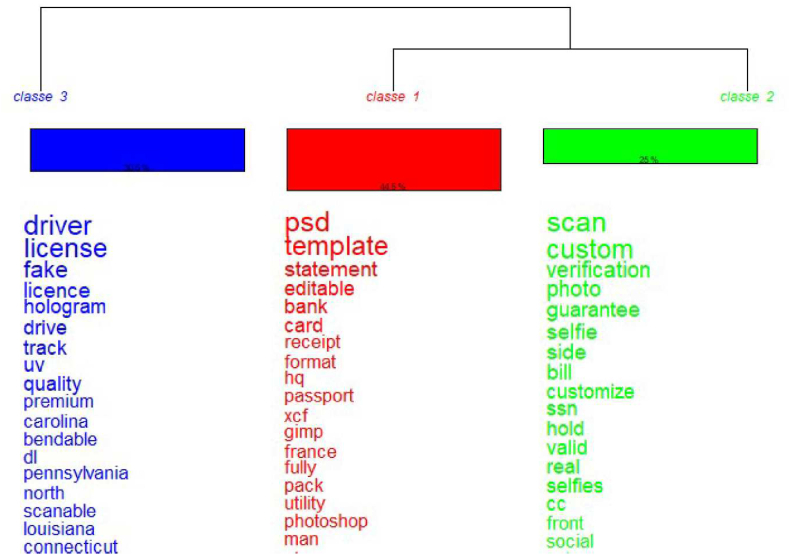
Dendrogram representing the distribution of the analysable forms between the three classes detected by the classification (N = 1187 ad titles analysed).

Three distinct classes have been found based on the title of the ads with the DHA. The dendrogram in Fig. 1 shows that the classes are quite balanced in terms of the percentage of forms: 44,5% for class 1, 25% for class 2, and 30,5% for class 3 (N = 1187 ads).

Class 1 gather terms linked to documents sold in digital format, with terms like “*psd*” (which corresponds to the Photoshop format), “*template*” (i.e. a base that can be modified by the user), or “*gimp*” (which refers to a tool for image edition like Photoshop). The presence of the term “*passport*” is linked to the presence of the expression “*passport psd template*” in 75 ads. The term “*card*” is also present, as well as many country names, which may be linked to advertisements offering passports for each particular country. Besides that, most of the terms are not specific to fake documents.

It is harder to find a main topic for the terms gathered in class 2. Nonetheless, terms linked to photos and scans seem to emerge. For instance, the term “*selfie*” corresponds to an image of a person holding an identity document. This type of photo is increasingly required in online authentication processes. The expression “*custom listing*” is also present. Custom ads are specific ads created for a specific client that are often deleted after the sale is made. It is usually a personalised ad without description as a result of a prior agreement between the seller and the customer [33]. Noticeably, the term “*passport*” is also present in this class. The term “*identity*” is present but is not directly linked to the term “*card*”.

For class 3, the main topic is driver's licenses. Ads for driver's licenses are more often than not linked to American driver's licenses (some American states even emerge as the main words). Some terms linked to security features like “*secu*”, “*hologram*”, “*uv*”, or “*holo*” are also frequently present in this class.

Combined with the dendrogram, the bag-of-word analysis reveals similarities between classes. In particular, the terms in common are “*passport*”, “*fake*”, “*license*”, and “*quality*".

**Fig. 2:**
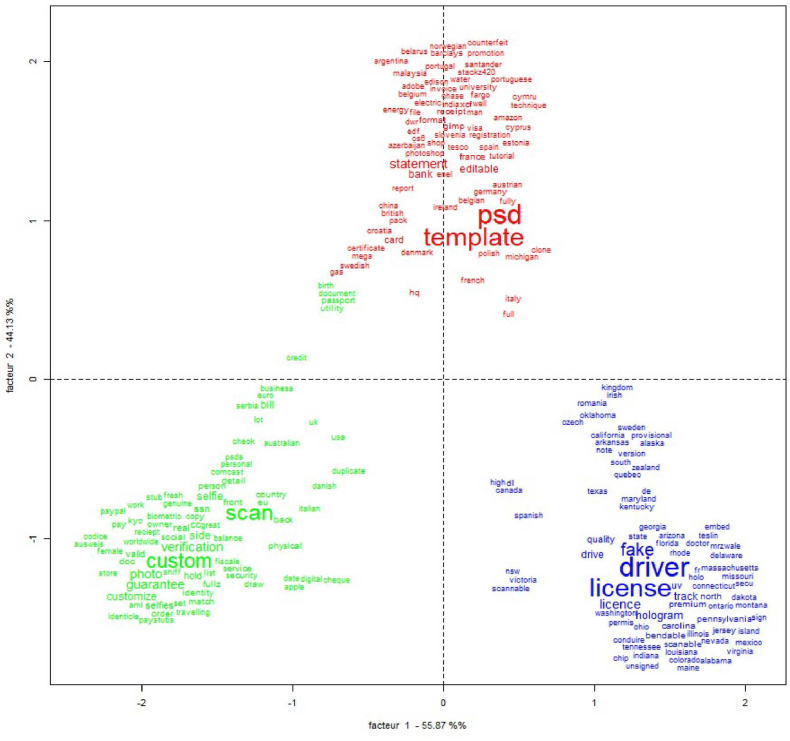
CFA on the classes identified from the DHA (class 1 in red, class 2 in green and class 3 in blue). (For interpretation of the references to colour in this figure legend, the reader is referred to the Web version of this article.)

The CFA (see Fig. 2), confirm that the three classes are well separated from each other. Class 3 is quite well isolated. This is particularly true for the American states, which seems to be very specific to this class.

Some terms like “*passport*” stand between class 1 and 2 because they appear in the text segment from the two classes. This can be explained by the fact that some ads propose *“passport scan”* in class 2, and “*passport psd template*” is one of the major n-grams from class 1.

#### 4.1.1 Similarity analysis

**Fig. 3:**
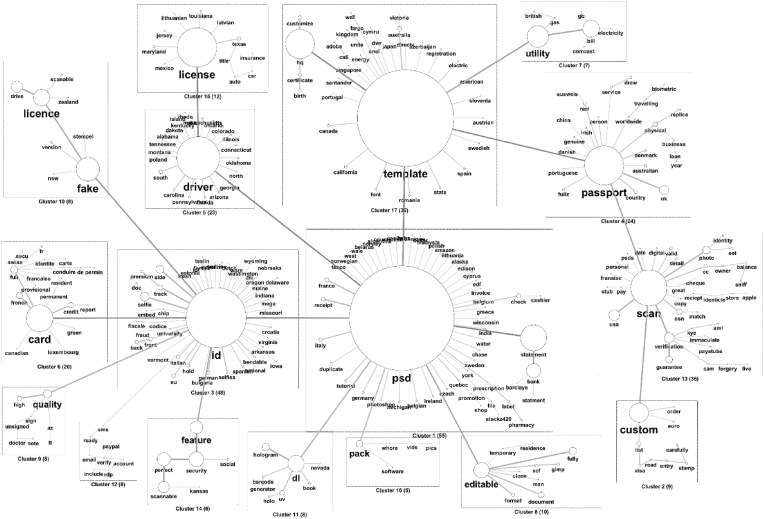
Similarity analysis, cooccurrence spanning tree, and clustering of the main terms (N = 322 forms analysed). The size of the nodes and width of the edges is proportional to the frequency of occurrences.

The analysis detects the terms that are most frequently used in the ad titles to describe the products and their relationships. The most frequent terms seem to match with the types of counterfeit documents (see Fig. 3): “*id*”, “*card*”, “*passport*”, “*driver*” and “*license*”. Certain terms are very often used together. For instance, the term “*id*” seems very central and rather generalist, as it leads to different types of documents, not only “*id cards*”. Moreover, the analysis leads to the detection of the different digital forms in which products can be found, like “*psd*”, “*template*”, and “*scan*”.

##### 4.1.1.1 Driver's licenses

A strong link is detected between both “*license*” and “*psd*”, with “*driver*” as the central term. This analysis highlighted many forms of expressions to nominate driver's licenses, including spelling differences (“*licence*”, “*drive*”) or diminutive forms (“*dl*”). Most of the terms linked to the two principal ones are American States, consistent with the result found during DHA analysis. Finally, the term “*dl*” is mostly associated with security features, like “*UV*” or “*hologram*”.

##### 4.1.1.2 ID cards

The term “*id*” is frequently linked to the words “*card*” and “*fake*”. The cluster centered around the term “*id*” is mostly composed of American states and country names. Another interesting thing is that the cluster containing the term “*card*” is also composed of French terms like “*conduire*” “*identité*” or “*carte*”. The “*id*” word is also frequently linked to the “*psd*” term.

##### 4.1.1.3 Passports

The term “*passport*” is also central and linked to 24 other forms. It is frequently linked to “*template*” and “*scan*”, which gives an indication of the type of counterfeiting. It is interesting to notice that the term “*physical*” is also linked to “*passport*”. We can also find “*biometric*” passports, which is indirectly related to “*passport*” (with “*world*” and “*travelling*” between them).

##### 4.1.1.4 Other types of documents

The similarity analysis also highlights other types of products categorized as fake documents, such as birth certificates (N = 16 and N = 17), utility bills (N = 119 and N = 96), bank statements (N = 82 and N = 147), or apple store receipts (N = 4, N = 6 and N = 21).

##### 4.1.1.5 Digital forms of documents

The first thing to notice is the strong link between “*psd*” and “*template*”, which is coherent with the observations that those two terms are often used together in the same texts and seems to be a very current format for digital documents. Passports also seems to be frequently linked to the term “*scan*”. Terms like “*gimp*”, “*editable*”, or “*xcf*” give other information about the digital documents format.

If digital forms seem to be central for fake documents, one term gives more insights about the context of their potential usage: the term “*selfie*”. It corresponds to photos showing a person, holding an ID document. Indeed, the digital transformation of services like neo-banking allows users digitally validate their accounts without any physical validation. Clients are requested to send a photo of themselves holding their ID document. Sometimes a piece of paper with the current date is also required in the picture. The identity control process is thus completely digital and might explain the appearance of new forms of illicit market for fake documents. In conclusion, the analysis shows that it is possible to detect specific kinds of fake documents that appear to be different from those described in the literature [13,14].

### 4.2 Comparing fake documents with other products

A DHA has been performed on every mixed corpus to see if the method can allow the discovery of new categories of products. For each class, we can distinguish a main topic that links all the words of the class together. In every mixed corpus, a specific category containing the forms linked to fake documents was also detected, except for the mixed corpus “*Fakedoc_drugs*”. This can be explained by the huge proportion of drug ads compared to fakedoc ads (85′262 and 1′321). This is also observed with the “*fakedoc_defense*” mixed corpus, where fake documents are predominant (1321 and 88). Observing the CFA generated successively to the analysis, it is possible to notice that, in two cases (“*Services*” and “*Online business”),* the class containing terms linked to fake documents are confounded with other classes. It can be explained by the fact that some terms are common among the products proposed in those categories, like “*card*” (which can fit with “*gift card*” or “*id card*”, for example). Moreover, the two subsections of fake documents were originally a part of the “*Services*” category, so it makes sense that the proposed products are close. For the online business section, it is possible to see that some terms are also semantically close. For example, this category contains a lot of “*bank drops*” (i.e. accounts that can be used for money laundering or illegal transfers) or credit cards. Terms linked to payment methods were also detected in the analysis of the fake document sections, such as “*paypal*”. (See Table 3)

**Table 3:** Number of classes obtained by CHD per corpus and distinction of a class related to false documents. N indicates the number of analysed ads for every corpus. The first ten words of each class found are also reported.

Corpus	Number of classes	Top 10 words in each class (by number of occurrences)
Fakedoc_onlinebusiness (N = 9813)	4	account; warranty; premium; porn; lifetime; extra; market; cheap; bonus; month
		hq; psd; template; card; id; scan; license; driver; passport; dl
		hq; usa; card; bank; cc; fullz; fresh; balance; email; verify
		database; record; hack; leacked; plaintext; million; dtabase; leak; voter; log
Fakedoc_forgeries (N = 2812)	4	psd; template; id; driver; license; passport; scan; hq; card; statement
		replica; perfect; shoe; vuitton; louis; lv; gucci; black; bag; dior
		series; gold; black; watch; rolex; box; pro; counterfeit; clone; max
		fakemoney; series; eur; test; pen; pass; uv; usd; version; stripe
Fakedoc_software (N = 3489)	4	pro; full; crack; program; macos; adobe; x64; window; pack; hack
		full; software; mac; source; tool; code; bitcoin; rat; android; stealer
		premium; porn; video; account; lifetime; movie; book; private; spotify; proxifier
		psd; template; id; driver; license; passport; scan; card; hq; statement
Fakedoc_services (N = 3436)	6	psd; template; id; driver; license; passport; statement; fake; utility; usa
		id; scan; passport; utility; custom; usa; quality; dl; high; bill
		complet; credit; full; uk; pack; list; delivery; real; utter; service
		card; hq; egift; pdf; restaurant; grill; pizza; italian; group; bar
		account; lifetime; premium; warranty; porn; quality; vpn; high; instagram; guarantee
		book; video; mastery; academy; market; figure; facebook; amazon; trade; dan
Fakedoc_defense (N = 1173)	3	id; driver; license; fake; licence; drive; quality; track; high; australia
		psd; template; passport; hq; card; statement; utility; editable; bank; fully
		passport; scan; card; utility; custom; bill; verification; usa; uk; selfie
Fakedoc_drugs (N = 78725)		gram; quality; ship; free; mdma; cocaine; pure; high; 5 g; ketamine
		quality; pill; mdma; high; top; xtc; mg; europe; dutch; import
		ship; pill; mg; xanax; sale; usa; duplicate; 10 mg; bar; price
		free; 5 g; uk; top; thc; indoor; sale; aaa; grade; haze
		ship; free; thc; 1 g; new; premium; fast; cannabis; g; day
All_sauf_drugs (N = 15844)	8	hq; card; usa; cc; full; bank; fullz; fresh; scan; email
		account; premium; warranty; hq; extra; market; cheap; bonus; month; access
		account; premium; warranty; porn; lifetime; extra; bonus; video; movie; include
		hq; psd; template; perfect; full; bank; id; scan; license; driver
		hq; card; egift; pdf; gift; money; save; lot; checker; code
		full; pro; pack; crack; complete; vpn; security; program; gold; adobe
		database; record; hack; leacked; plaintext; million; dtabase; leak; voter; log
		perfect; replica; shoe; high; quality; vuitton; louis; lv; gold; gucci

### 4.3 Detecting fake documents in other sections

The major interest of using a concordance table is to determine if it is possible to detect bad categorisation of fake documents in other sections. For this analysis, the first five words of each class (by number of occurrences) found from the DHA analysis of the “*listing_fakedoc*” corpus were searched in the “*listing_all_without_drugs*” corpus. It seems important to notice that the terms studied in this analysis have been selected according to their number of occurrences in the corpus. They are thus not necessarily specific to the field of fake documents. Then, every category different from the two fake document subsections (physical/digital) was identified. Table 4 shows all the detected categories.

**Table 4:** Presence/absence of the term in a category other than “Fake Document (Physical)" or “Fake Document (Digital)".

	Class 1					Class 2					Class 3							
	psd	template	passport	hq	statement	passport	scan	utility	custom	bill	driver	license	fake	licence			dl	
Online business, other fraud related	x	x	x	x	x	x	x	x	x	x	x	x			x		x	
Online business, SSN/DOB/OtherPII	x	x	x	x		x	x	x	x	x	x	x				x		x
Online business, drops others		x							x									x
Online business, dumps		x	x			x	x		x		x	x				x		
Online business, card and CVV			x	x		x	x	x	x	x		x				x		x
Online business, various logins			x	x		x				x	x	x				x		
Online business, corporate intel				x							x							
Online business, drops bank				x			x		x	x		x						x
Online business, bank login				x			x			x	x					x		x
Services, carding	x	x					x		x		x	x						x
Services, Hosting										x								x
Services, Operational management							x											
Services, Other services			x	x		x	x	x	x	x	x	x				x		
Services, social engineering		x	x			x												
Services, VPN				x												x		
Services, SOCKS				x					x									
Services, security												x						
Forgeries/counterfeit, currency	x	x							x	x			x					
Forgeries/counterfeit, other forgeries			x			x		x	x				x					
Forgeries/counterfeit, electronics									x									
Forgeries/counterfeit, watches													x					
Software, other software		x					x	x	x		x	x	x			x		
Software, commercial software				x				x				x				x		
Software, botnet and malware				x														
Software, exploit kit							x											
Software, security software							x		x			x				x		
Defense counter intel, frequency scanner/bug detector							x											
Defense counter intel, operational security									x									
**Total per word**	4	8	8	13	1	8	13	7	15	9	9	12	4			11		8

Twenty-eight categories containing the first five words of our “*fake document*” classes have been identified. The term with the highest diversity is “*custom*”, present in 15 categories. As previously described, this can be explained by the particular usage of this term within the cryptomarket ecosystem. “*passport*” is nevertheless present in 8 other categories. The term “*statement*” is the one with the least other categories.

The specificity of the terms can also be analysed with the proportion of their occurrences in the “*fakedoc*” corpus compared to their total number of occurrences (Table 5).

**Table 5:** Proportion of occurrences in the “fakedoc” corpus compared to the total corpus (except drugs) (N = 15844 titles for the “listing_all_without_drugs” corpus and N = 1187 titles for the “fakedoc” corpus).

	Word	Total number of occurrences	Number of occurrences in the “listing_fakedoc” corpus	Proportion
Class 1	psd	648	562	87%
	Template	616	516	84%
	Passport	311	205	66%
	Hq	1265	161	13%
	Statement	148	144	97%
Class 2	passport	311	205	66%
	Scan	399	199	50%
	Utility	147	119	81%
	Custom	201	105	52%
	bill	183	93	51%
Class 3	driver	321	236	74%
	License	343	217	63%
	Fake	219	130	59%
	Licence	162	91	56%
	dl	280	81	29%

The terms with the highest rate of occurrences in the “*listing_fakedoc*” corpus are “*statement*” (97%), and “*psd*” (87%), which is consistent with the previous results, in particular concerning the most common format of selling. The terms with the lowest rate of specificity are “*hq*” (13%) and “*dl*” (29%). “*hq*” is an abbreviation of “*high quality*”, which is an expression that can be used in many other contexts than fake documents. “*dl*” can be translated as “*driver's license*”, but also “*download*”.

### 4.4 Grouping sellers

#### 4.4.1 Based on the ad titles

Seven successive CFA were performed, during which 19 outliers were removed. Outliers ads are mostly written in other languages, like French or German. Products like Netflix accounts, Walmart receipt, Apple store subscriptions, and biometric passports and visas were also detected as very specific selling activities related to outliers.

**Fig. 4:**
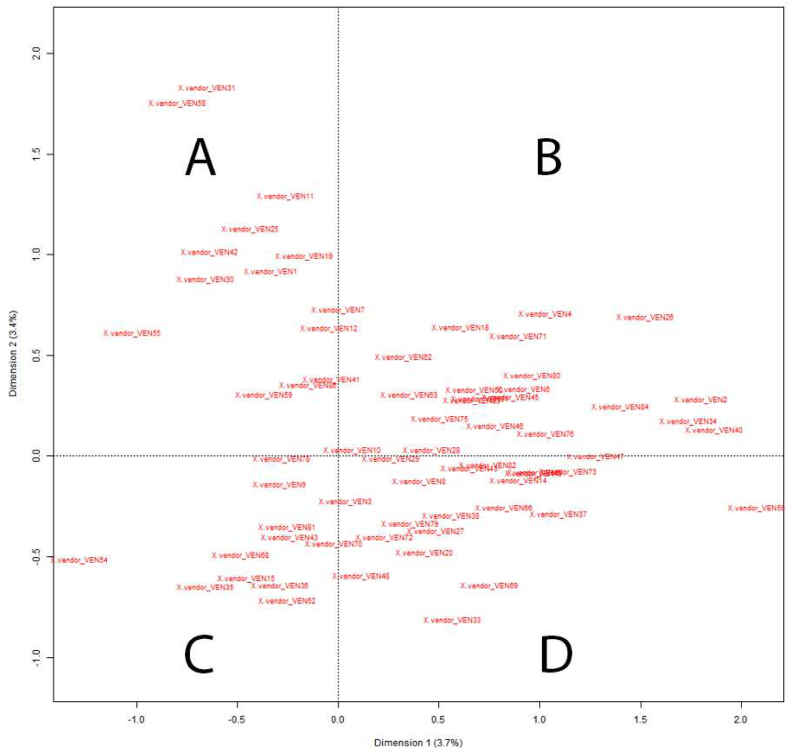
Final CFA obtained as a result of successive analyses of the specificities of sellers of false documents based on ad titles (N = 67 sellers present on the CFA at the last analysis).

Fig. 4 shows the result of the last CFA, where no obvious outliers remain visible. Each square on the graph represents a group according to the dimensions selected by the algorithm.

By consulting the catalogue of the vendors from each group, we were able to extract a main topic of products for each group:

- Group A: physical fake documents. This is the group with the fewest vendors, which is consistent with the other analysis showing that digital fake document are more common than physical ones.
- Group B: “*scan*”, “*custom*”, “*selfie*” are the most common products in this group. This result is also consistent with class 2 of DHA analysis discussed earlier.
- Group C: “*psd template*”. Given the omnipresence of this digital form of products on the market, it is not surprising to find a group comprising mainly the sellers of this type of document.
- Group D: “*pack*” and other products. This group is more difficult to define in terms of products sold. The term “*pack*” is present in an important part of the ads, but this group contains some diversity.

We can see that some vendors are really close to the axis, which can be explained by the proximity of their offer with vendors from other groups. For example, vendor 10 (group A) is really close to group C, and by looking at his catalogue, we can see that his offer is mostly composed of “*psd template*” ads, among other products.

The contribution of each group can be visualised in Table 6. The distribution of the vendors through the groups is balanced.

**Table 6:** Contribution of each group and outliers to the total number of vendors (N = 86).

Group	Headcount	Percentage of total
1	15	17,4%
2	20	23,3%
3	13	15,1%
4	19	22,1%
Outliers	19	22,1%
**Total**	**86**	**100,0%**

#### 4.4.2 Based on their profile

The same analysis was performed using the vendor's profile textual description. This analysis revealed two major issues: the first is that 17 vendors didn't have any written profiles, so they can't be taken into account. The second one is that, after six successive analyses, 29 vendors were excluded as they appear as outliers, resulting in a total of 46 vendors (53% of the total) that weren't analysed. The reading of the profiles did not reveal any insights into the groups formed with the remaining sellers. The profiles of the outliers did not reveal anything conclusive either to explain their exclusion from the others.

## 5 Conclusive discussion

*Is it possible to set up a classification of fake documents using textual data?* DHA analysis led to a classification of fake documents and highlighted other types of documents than fake identity documents described in the literature, which distinguish between three main categories: passports, ID cards and driver's licenses. The highlighting of other products like utility bills, and bank statements, but also a novel category related to “selfies”, shows a bigger diversity in the market than expected. The similarity analysis is informative on the most common format of selling the products: the “*psd template*” format. Based on the observation that driver's licenses are mostly linked to American state names, it can be hypothesised that the demand for this kind of document is higher. Indeed, driver's licenses are much more used to check identity in the USA than the id card or passport. The discovery of the selfie brings to light new issues concerning identity control on the internet. Indeed, today, many sites require a photo of the user holding an ID to access their services. The availability of these selfies, therefore, offers a new way of evading these controls.

However, during DHA, IRaMuTeQ showed its first limits. The term “*id*” was absent from the analysis. The assumption made about this fact is that term was systematically contained in the texts that weren't taken into account. Another hypothesis was suggested by Ref. [34]. She suggests that the software does not take every form as “*full forms*”. The major problem is thus that the operator has no control of the forms or texts analysed, which is a real issue from a forensic point of view. To test the hypothesis, the term “*id*” was replaced with “*identity*” in the corpus. After another DHA, the term “*identity*” appeared in the class associated with driver's licenses, with a higher number of occurrences. This finding raises the hypothesis of small words being excluded just like stop words. They may not be taken into account because of their size. However, terms such as “*hq*” and “*dl*” were taken into account in the analysis. This observation led to the fundamental methodological proposition recall by Ref. [12]: “*back to the text*”. As it helps to identify these gaps induced by analyses over which the operator's control is limited, it compensates for the “black box” effect inherent in some algorithms. In our study, this problem appears to be specific to DHA analysis. The modification of the corpus made in the test may not be a viable solution, because, depending on the context, this action could be perceived as a modification of the textual trace.

*Can fake documents be distinguished from other products?* In most cases of mixed corpora studied, it was possible to distinguish a specific theme for the classes found with DHA on mixed corpus, and to get a separate class containing forms linked to fake documents from other classes. The main issue for the comparison is the variation in the sizes of the corpus. If one of the two categories used to create the mixed corpus has many more texts than the other, the second one is hardly detected. Following this, the concordance table led to the detection of forms that can be used in different contexts and wrong categorisations of fake documents. Freeing oneself from the sections used by the sellers to select the product to analyse is a key issue for analysing online marketplaces. This was not the main aim of this study, but results show the interest of the tested approaches to evaluate the results of fully automated IA approaches like deep-learning ones.

*Can sellers of fake documents be grouped based on the textual data from the advertisements?* Four main groups of vendors were detected. Globally, an important proportion of digital fake documents are observed compared to physical ones. The effort required for making physical documents and the ease of transferring digital documents may explain this result. Indeed, the manufacture of fake documents requires know-how, equipment and materials to produce a document that is of satisfactory quality. There is also consistency between results found with the products and with the vendors, which might signify a certain degree of specialisation. The main issue of this analysis is the exclusion of the outliers during the successive CFA. Indeed, this part of the process is based on a visual analysis of the graphs. The operator decides which vendor is an outlier based on its graphical distance from the main group. In that case, there was always a compact group in the centre, so it was easy to determine the outliers.

*Is it possible to find groups of sellers from the analysis of their profiles?* This analysis suffers from the subjectivity required for the exclusion of the outliers. Contrary to the corpus of ads titles, the distributions obtained after the successive specificity analysis for the vendor's profiles were more shattered. This led to the exclusion of 29 vendors (34%). Knowing that 17 vendors have no profile, 53% of vendors were considered in the analysis. Vendors' profiles should thus be considered with cautiousness, and further analysis is required to evaluate their informative content. It was indeed impossible to identify the main topic for the groups formed. This can be explained by the fact that every vendor chooses to write whatever they want in their profile, and it doesn't necessarily have a link with what they sell. It could be interesting to try the method with a corpus of vendors of other types of products to see if it is an inherent problem for vendors of fake documents.

Globally, several steps of the methodology used required manual work, which leads to a certain risk of error. For example, in the concordance table analysis, it would have been difficult to estimate the number of products listed outside the fake document categories for each term studied, due to the high proportion of occurrence of each word. IRaMuTeQ did not allow for an automatic numerical estimate. The size of the corpus is also a limitation for some analyses, such as product classification. This method requires the assess the construction of the corpus itself, in order to ensure that all forms are taken into account. Finally, some limitations come from the software used. Indeed, IRaMuTeQ is an easy-to-use software provides good results for exploratory analysis and relevant global information. However, it does not allow us to go deeper into the details of the data, at least not in an automatic way. Furthermore, the operator has little control over the forms used. It could therefore be interesting to place it in sequence with other techniques, where it would allow an initial sorting to be carried out before continuing with more elaborated methods and tools.

The analysis of words as a trace in the judicial context is an issue that still raises many questions. Indeed, words are more often than not considered subjective and sensible to a lot of variation and interpretation, an aspect that statistical methods tend to mitigate. But the potential of these methods during investigation and for intelligence purposes appears to be very high. This research work is intended to be a starting point and, above all, an open door to explore how the statistical analysis of textual data might help to answer crime analysis questions.

## Declaration of competing interest

No conflict of interest.

## Footnotes

1 https://darknetone.com/market/white-house-market-whm/

2 http://iramuteq.org/

3 https://www.yworks.com/products/yed
